# Development of a Xeno-Free Substrate for Human Embryonic Stem Cell Growth

**DOI:** 10.1155/2015/621057

**Published:** 2015-03-16

**Authors:** Hailin Zhu, Jinliang Yang, Yuquan Wei, Harry Huimin Chen

**Affiliations:** ^1^State Key Laboratory of Biotherapy, Sichuan University, Chengdu, Sichuan 610041, China; ^2^StemEasy Biotech, Ltd., Jiang Yin, Jiangsu 214400, China

## Abstract

Traditionally, human embryonic stem cells (hESCs) are cultured on inactivated live feeder cells. For clinical application using hESCs, there is a requirement to minimize the risk of contamination with animal components. Extracellular matrix (ECM) derived from feeder cells is the most natural way to provide xeno-free substrates for hESC growth. In this study, we optimized the step-by-step procedure for ECM processing to develop a xeno-free ECM that supports the growth of undifferentiated hESCs. In addition, this newly developed xeno-free substrate can be stored at 4°C and is ready to use upon request, which serves as an easier way to amplify hESCs for clinical applications.

## 1. Introduction

Since the first hES cell line derived in 1998 [1], hESCs have been routinely cultured on inactivated CF-1 mouse feeder cells (MEFs). Maintaining the undifferentiated growth of hESCs on feeder cells is time-consuming and costly. The number of feeder cells must be carefully calculated as either higher or lower density feeder cells to promote hESCs differentiation. To simplify this process, Xu et al. [2] developed Matrigel as a substrate to culture hESCs. Matrigel is a solubilized basement membrane preparation extracted from the Engelbreth-Holm-Swarm (EHS) mouse sarcoma [3]. Matrigel is the most widely used substrate in a feeder-free culture system. However, both the mouse feeder cells and Matrigel are not xeno-free substrates. The risk of unknown nonhuman components limits their application in clinical practice.

For clinic applications, several different kinds of xeno-free substrates have been developed in recent years, such as human feeder cells [4, 5], vitronectin [6–8], laminin [9], RGD peptide [10], and human placenta derived ECM [11]. Some of these are not cost effective and cannot be produced in large scale, while others cannot maintain the hESCs growth for a long time.

MEFs are known to express Neu5Gc (N-glycolylneuraminic acid) on their surface [12]. Additionally, Stelling et al. [13] fixed MEFs using 70% ethanol as a coating substrate to support hESCs. They observed that ethanol-fixed MEFs matrix decreased the levels of Neu5Gc in hESCs by 30% but still maintain the hESCs adherence, growth, and pluripotency. Our previous report modified their methodology and fixed MEFs using 90% methanol. As a result, hESCs maintained pluripotency on these methanol fixed MEFs for 10 passages, but this substrate was not xeno-free and hESCs did not attach well after 10 passages [14].

In our current study, we lysed human umbilical cord stromal cells (hUCSCs) to obtain xeno-free ECM as coating material and then fixed them with 80% methanol on the plate. After 25 passages hESCs still grew on the fixed ECM; we compared the morphology of hESCs cultured on this fixed ECM with those on inactivated live MEFs or Matrigel and checked the hESCs karyotype, Alkaline phosphatase (ALP) activity, embryonic specific and pluripotency related gene expressions, and teratoma formation* in vivo*. Additionally, we checked the stability of the coated plates after they were stored at different conditions and for multiple time points.

## 2. Materials and Methods

### 2.1. Isolation and Culture of Human Umbilical Stromal Cells

Human umbilical cords were obtained from healthy full-term infants. Women donating the infant tissues signed the inform consent, and the project was approved by an independent review board from Jiangsu Institute of Parasitic Diseases. Human umbilical cords were handled under sterile condition and washed thoroughly with PBS. The arteries and veins were removed to obtain Wharton's jelly. Then, Wharton's jelly was chopped into small pieces and cultured in DMEM/F12 supplemented with 20% FBS for 2-3 weeks. After the first passage, the cells were maintained in DMEM/F12 supplemented with 10% FBS.

### 2.2. Preparation of Methanol Fixed Xeno-Free ECM Coated Plate

Human umbilical stromal cells (hUCSCs) were plated at a density of 2 × 10^4^/cm^2^ and cultured in DMEM/F12 (Hyclone) supplemented with 10% FBS for 4-5 days to reach complete confluence. After rinsing with PBS, they were incubated in 0.3% DOC in 10 mmol/L Tris-HCl + 10 mM EDTA, pH 8.0 for 5 minutes at room temperature, rinsed four times with PBS, fixed with methanol for 10 minutes, dried, and stored at 4°C.

### 2.3. hESCs Culture and Passage

The hESC cell line X-01 [15], provided by the Stem Cell Bank, Chinese Academy of Sciences, was cultured on irradiated inactivated CF-1 MEF in DMEM/F12 supplemented with 20% Knockout Serum Replacement (KSR, Invitrogen), 200 mM glutamine, 55 mM 2-mercaptoethanol, 1% nonessential amino acids (all from Invitrogen), and 8 ng/mL FGF-2 (Peprotech), for long term maintenance. hESCs were cultured on methanol fixed xeno-free ECM plate with either mTeSR1 (STEMCELL) [16] or TeSR-E8 medium (STEMCELL) [17].

hESCs grown on methanol fixed xeno-free ECM were passed by Dispase (1 mg/mL) for 5 minutes at 37°C or by Gentle Cell Dissociation Reagent (STEMCELL) for 5 minutes at room temperature, followed by cell detachment with a scraper.

### 2.4. AP Staining

Alkaline phosphatase (ALP) activity was detected using the ALP detection Kit (Sidansai) according to the manufacturer's protocol. The stained cells were imaged with an Olympus IX71 fluorescence microscope.

### 2.5. Immunofluorescent Staining

To detect the pluripotency related surface marker SSEA4, Tra-1-60, Tra-1-81, and hESCs (passage 25) were fixed with 4% paraformaldehyde in PBS for 30 minutes and incubated with primary antibody overnight at 4°C and then incubated with secondary antibody for 1 hour at room temperature. To detect the pluripotency-related nuclear marker Oct4, Nanog, and Sox2, the fixed cells were permeabilized with 0.1% Triton X-100 and then followed with desired first and secondary antibodies incubations and washing. After antibody staining, cells nuclei were stained with DAPI and imaged with Olympus IX71 fluorescence microscope.

### 2.6. Quantitative Real-Time PCR

To compare the pluripotent gene expression between hESCs culture on Matrigel and methanol fixed xeno-free ECM, total RNA was extracted from undifferentiated hESCs using TRIzol (NJBIOSKY) and transcribed into cDNA using oligo(dT)16 and M-MLV Reverse Transcriptase Kit (TIANGEN). For quantitative real-time PCR, the primer sequences were as follows: for Oct4: F: CTTGCTGCAGAAGTGGGTGGAGGAA; R: CTGCAGTGTGGTTTCGGGCA. Sox2: F: CGGCAACCAGAAAAACAGC; R: CCGACAAAAGTTTCCACTCG. Nanog: F: AGGCAAACAACCCACTTC; R: CTTCTGCGTCACACCATT. CAC: F: GGAGTTATGGTGGGTATGGGTC; R: AGTGGTGACAAAGGAGTAGCCA. AFP: F: GCTGGATTGTCTGCAGGATGGGGAA; R: TCCCCTGAAGAAAATTGGTTAAAAT. Samples were used for 40-cycle PCR in SYBR Green Master Mix (NJBIOSKY). The conditions for PCR reactions were as follows: denaturation at 95°C for 3 minutes, 40 cycles of denaturation at 95°C for 30 seconds, annealing at 55°C for 20 seconds, and extension at 72°C for 20 seconds. GAPDH was used as internal control. The equation describing the plot of threshold cycle, Ct, versus log concentration was used to determine relative amounts of mRNA in experimental samples. From the Ct values, the relative transcript concentration was calculated and normalized to that of the internal control.

### 2.7. Karyotype Analysis of hESCs

To analyze the karyotype of the hESCs cultured on the fixed xeno-free ECM, hESCs (X-01, passage 25) were incubated with 0.1 *μ*g/mL of colcemid (Sigma) for 3 hours, then trypsinized and incubated with 75 mM KCl for 15 minutes at 37°C, and fixed with methanol/glacial acetic acid 3 : 1 (v : v) solution. G-band staining was used to visualize the chromosomes, with 20 metaphases counted and scored for karyotyping.

### 2.8. Analysis of Differentiation Ability of hESCs* In Vivo*

To analyze the ability of hESCs to differentiate into three layers* in vivo*, hESCs cultured on methanol fixed xeno-free ECM (passage 25) were digested; clumps of hESCs (2 ~ 3∗10^6^) were injected into the rear leg muscle of NOD-SCID mouse. Three months after the injection, the NOD-SCID mouse was sacrificed and the mass was dissected. The mass was fixed in Bouin's fixative and embedded in paraffin and then sliced into pieces in 5 *μ*m thickness. After dewaxing, the sections were stained with hematoxylin and eosin. The stained sections were examined histologically.

## 3. Results

### 3.1. Methanol Fixed Xeno-Free ECM Supports the Growth of hESCs

**Figure 1:**
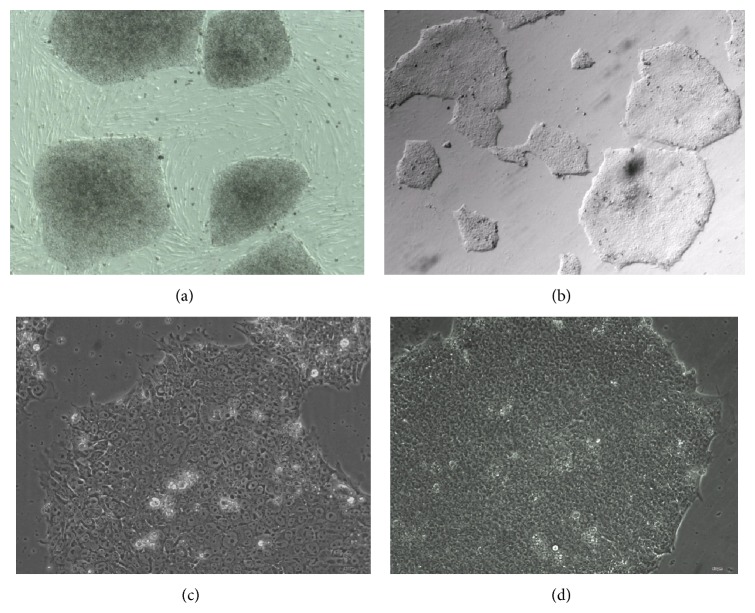
hESCs were grown on irradiated feeder cells, Matrigel, or methanol fixed xeno-free ECM. (a) hESCs were grown on irradiated MEF feeder cells in KSR complete medium (10x); (b) hESCs were grown on fixed xeno-free ECM in TeSR-E8 (10x); (c) hESCs were grown on Matrigel in TeSR-E8 (20x); (d) hESCs were grown on fixed xeno-free ECM in TeSR-E8 (20x).

**Figure 2:**
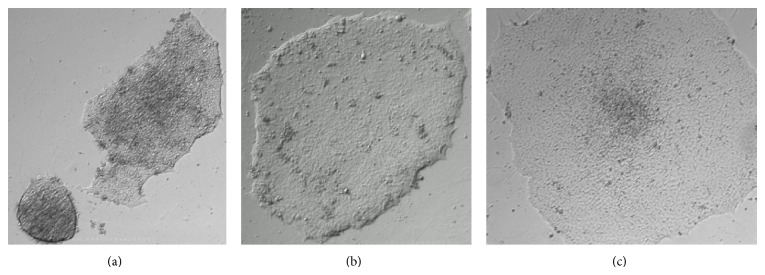
hESCs were grown on nonfixed ECM or methanol fixed xeno-free ECM in mTeSR1. (a) hESCs were grown on nonfixed xeno-free ECM in mTeSR1; (b) hESCs were grown on 80% methanol fixed xeno-free ECM in mTeSR1; (c) hESCs were grown on 100% methanol fixed xeno-free ECM in mTeSR1.

**Figure 3:**
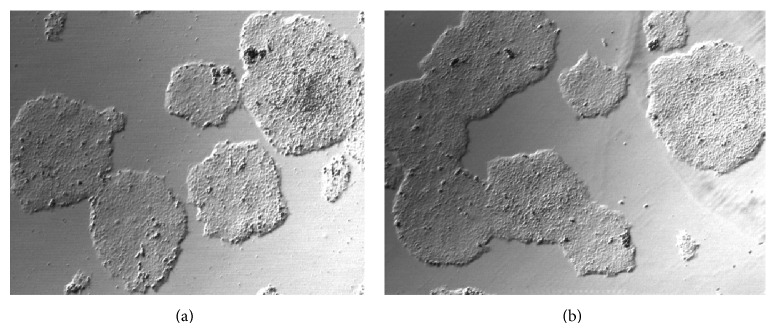
After 1 week or 6 months of storage at 4°C, no significant reduction in supporting activity was observed. (a) hESCs were grown on fixed xeno-free ECM coated plates that had been stored at 4°C for 1 week (10x); (b) hESCs were grown on plates that had been stored at 4°C for 6 months (10x).

hESCs can be cultured on methanol fixed xeno-free ECM in either mTeSR1 or TeSR-E8 medium. We observed that the concentration of methanol has an impact on the morphology of the colonies. When the colonies grew on nonfixed ECM, they packed tighter than when grown on live feeder cells (Figures 1(a) and 2(a)). As the concentration of methanol increased, the colonies became more dispersed (Figure 2). hESCs grown on 80% methanol fixed xeno-free ECM had a condensed morphology, which was similar to colonies grown on live feeder cells; however hESCs grown with 100% methanol fixation appear to be flat. We also checked the stability of the coating material after the plates were stored in various conditions and for different periods of time. The hESC supporting activity of fixed xeno-free ECM remained the same after it had been stored at 4°C for up to 6 months (Figure 3).

### 3.2. hESCs Maintained Pluripotency after They Grew on Methanol Fixed Xeno-Free ECM Coated Plate for 25 Passages

**Figure 4:**
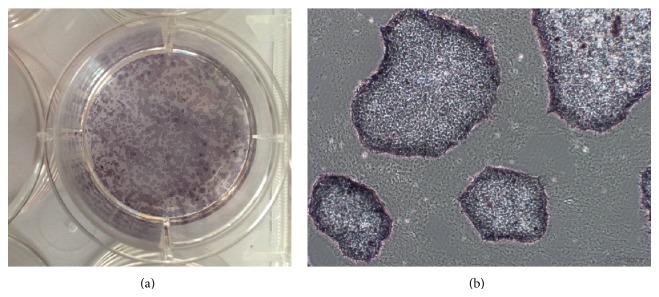
hESCs cultured on fixed xeno-free ECM at passage 25 were positive for AP. (a) Overview appearance of hESC colonies with AP staining on 6-well plate; (b) morphology of hESCs under the microscope after AP staining (10x).

**Figure 5:**
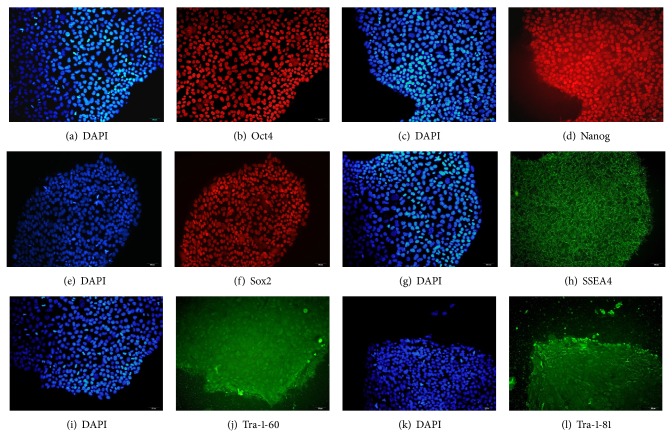
Detection of specific markers of hESCs cultured on fixed xeno-free ECM after 25 passages. ((a), (c), (e), (g), (i), and (k)) hESCs nuclear staining with DAPI; ((b), (d), (f), (h), (j), and (l)) hESCs with staining of Oct4, Nanog, Sox2, SSEA4, Tra-1-60, and Tra-1-81, respectively.

**Figure 6:**
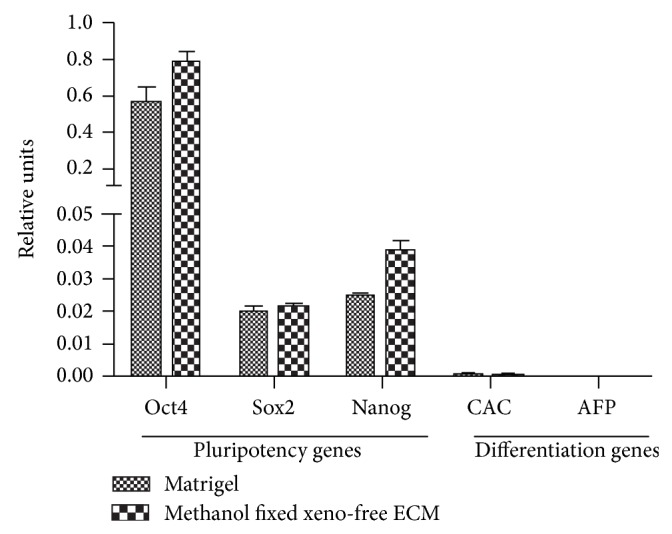
Quantitative PCR analysis of pluripotent gene expression in hESCs maintained for 25 passages on Matrigel or methanol fixed xeno-free ECM.

**Figure 7:**
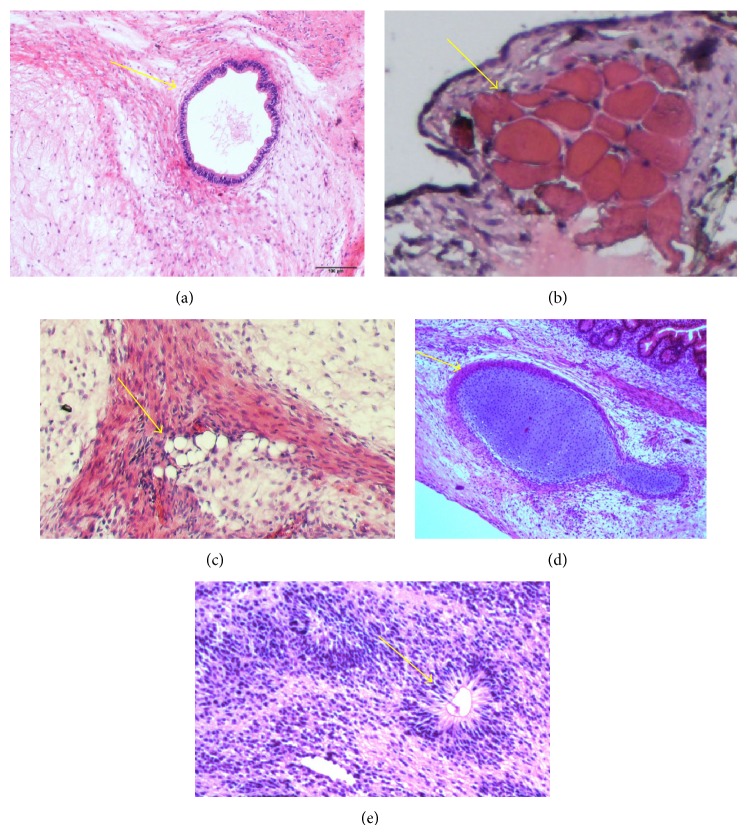
HE staining showed the teratoma had all three embryonic germ layers. (a) Endoderm (glands); ((b), (c), and (d)) mesodermal tissues of muscles, adipose, and cartilage, respectively; (e) ectoderm (neuroepithelium).

After 25 passages on fixed ECM coated plates, hESCs retained the ability of staining positive for AP (Figure 4). Additionally, these cells remained positive for all 6 pluripotency markers, including Oct4, Nanog, Sox2, SSEA4, Tra-1-60, and Tra-1-81 (Figure 5). At mRNA level, those cells continued to express pluripotency markers at the levels equivalent or superior to those detected in hESC grown on Matrigel. The early differentiation genes, CAC and AFP, were undetectable in both culture conditions (Figure 6). Moreover, they were able to form teratomas* in vitro*. HE staining showed that the teratoma had three embryonic germ layers (Figure 7).

### 3.3. hESCs Maintained the Normal Karyotype on Methanol Fixed Xeno-Free ECM

**Figure 8:**
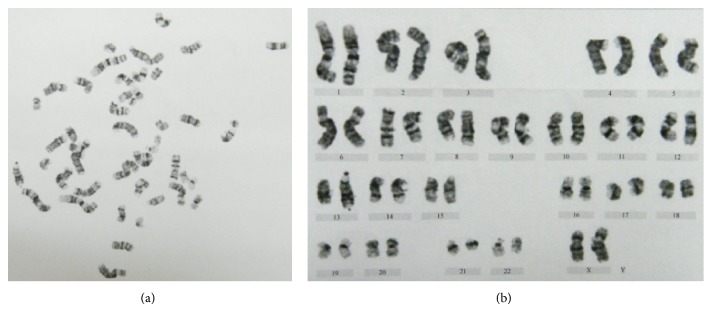
Karyotype analysis of hESCs grown on fixed xeno-free ECM after 25 passages.

The karyotype is an important indicator of the stability of hESCs culture system. In an unstable system, hESCs may acquire an abnormal karyotype after long passages in culture. We tested the karyotype of hESCs after 25 passages over 4 months cultured on methanol fixed xeno-free ECM; G-band staining of chromosomes showed the normal karyotype (Figure 8).

## 4. Discussion

The culture system for hESCs has been modified in recent years, from standard culture on MEFs or on human feeder cells to feeder-free culture system. It has been reported that the most reproducibly successful feeder-free substrate for hESCs is Matrigel. However, Matrigel is a reconstituted basement membrane made from a mouse EHS tumor. This preparation risks transmitting animal proteins, which prevent hESCs from clinical applications in humans. Ultimately, a xeno-free coating substrate combined with a chemical-defined medium is needed for clinical-grade hESCs.

In this study, we focus on developing a ready-to-use feeder-free and xeno-free substrate as coating material for long-term culture of hESCs. Compared to Matrigel and other purified ECM coating materials, this substrate is naturally derived from hUCSCs, and therefore, it contains only components of human origin. Moreover, there are no ethical concerns when using hUCSCs, and they can be passed for more than 15 passages [18]. This indicates that ECM derived from hUCSCs has the potential to be used for hESCs amplification in the clinical practice.

In our previous report, we improved the methods of Stelling et al. [13] by developing methanol fixed MEFs as a new substrate for culturing hESCs [14]. We observed that hESCs are maintained as densely packed colonies on the methanol fixed MEFs, but after a few passages they did not attach well from the feeders. It is possible that the fixed feeder cells perturb the ECM, which is an important element for the attachment of hESCs. To expose the ECM for hESC attachment, in this study, we lysed stromal cells from human umbilical cord. Although live hUCSCs have been reported as alternative feeder cells for culturing hESCs [18], we are the first one to lyse and fix hUCSCs on the plate for hESC culture. Furthermore, we have tried to follow the research of others to lyse additional human feeder cells, such as human placenta mesenchymal cells [19] or human decidual mesenchymal cells [20]; however, after decellularization, their ECM cloth structures could not remain intact as the ECM from hUCSC does.

We optimize the fixation conditions and found that the ECM fixed by 80% methanol maintains ECM cloth structures and supports hESC growth similar to that of live feeder cells. Moreover, these methanol fixed xeno-free ECM plates are stable and can be stored at 4°C for a long term (>6 months).

Aside from the ECM, the culture media must also be free of any animal components. In this study, we choose a chemical-defined medium TeSR-E8. To test the stability of the xeno-free culture system, hESCs were cultured on fixed ECM combined with TeSR-E8. After 25 passages in this system, hESCs maintain the same morphology as when cultured on live feeder cells; G-banding analysis of the cells shows a normal karyotype; immunofluorescence staining shows that the expressions of Oct4, Nanog, Sox2, SSEA4, Tra-1-60, and Tra-1-81 are positive; quantitative real-time PCR shows that the hESCs continued to express pluripotency markers at levels equivalent or superior to those grown on Matrigel; the cells form teratomas when transplanted into immunocompromised mice with evidence of all three germ layers.

Additionally, there is no difference when X-01 cells were cultured on our new substrate compared with those cultured on Matrigel when using mTeSR1 media. However, when using TeSR-E8 medium, X-01 cells can not survive on Matrigel (data not shown) but grew well on our ECM substrate. We also have tried other hESC cell lines, such as H1 and H9; they grow well in mTeSR1or TeSR-E8 on either Matrigel or our new substrate (data not shown). Since the formulation of TeSR-E8 is much simpler than mTeSR1, it is possible that our new substrate contains certain components that are essential for X-01 growth in TeSR-E8 medium.

Considering the source of hUCSCs is limited, we are planning to immortalize those cells and make a stable cell line from them. It would be relatively easier and more efficient to have the ECM from such cell line.

In conclusion, we have developed an efficient method to obtain xeno-free ECM as coating material for hESC amplification. Our data suggest that this ECM can be used with chemical-defined medium for long-term culture of hESCs. Furthermore, it is possible that this methodology can be used with other defined media to establish a completely xeno-free system to amplify hESCs for clinical applications.
